# Effects of pepper–mung bean intercropping on pepper productivity, nutrient accumulation, and rhizosphere microbial communities under continuous cropping

**DOI:** 10.3389/fpls.2026.1987577

**Published:** 2026-10-30

**Authors:** Shaoli Zhang, Peipei Chang, Jingjing Wang, Tengfei Li, Youping Wang, Meimei Han, Luqi Zhang, Qingqing Duan, Fangjing Hua, Zikun Zhang

**Affiliations:** Dezhou Academy of Agricultural Sciences, Institute of Horticultural Crops, Dezhou, Shandong, China

**Keywords:** continuous cropping, microbial community structure, pepper productivity, pepper–mung bean intercropping, Rhizosphere microbiome

## Abstract

Continuous monocropping can impair sustainable pepper (*Capsicum annuum* L.) production by disrupting soil nutrient availability, microbial communities, and biological functioning. Legume-based intercropping offers a potential strategy to alleviate these constraints, but its effects in continuously cropped pepper systems remain poorly understood. Here, a field experiment was conducted in a long-term continuously cropped pepper field to evaluate the effects of mung bean intercropping on pepper growth and yield, nutrient accumulation, rhizosphere soil properties, and microbial communities. Compared with pepper monocropping (MP), pepper–mung bean intercropping (IP) significantly increased average fruit weight and pepper yield, but did not affect plant height, stem diameter, or fruit number per plant. IP altered the accumulation and distribution of N, P, and K among pepper organs, particularly in reproductive tissues. In the rhizosphere, IP increased available N, P, and K and urease, dehydrogenase, and catalase activities, while total N, P, and K remained unchanged. Amplicon sequencing revealed significant differences in bacterial and fungal community composition between MP and IP. At the fungal genus level, *Bisifusarium* and *Neocosmospora* were enriched under MP, whereas *Conocybe* was enriched under IP. IP also increased the relative abundances of several bacterial taxa, particularly *Vicinamibacterales*, *Vicinamibacteraceae*, *KD4-96*, and *Rokubacteriales*. Several IP-enriched taxa were positively correlated with rhizosphere nutrient availability, soil enzyme activities, and pepper yield-related traits. Overall, mung bean intercropping was associated with improved rhizosphere nutrient availability and enzyme activities, together with substantial restructuring of bacterial and fungal communities, and may therefore contribute to improved pepper productivity under continuous cropping.

## 1 Introduction

Pepper (*Capsicum annuum* L.) is one of the most economically important vegetable crops worldwide and is widely cultivated for fresh consumption and processing (Hernández-Pérez et al., 2020). However, intensive vegetable production often involves repeated cultivation of the same or closely related crops, particularly where limited arable land constrains conventional crop rotation (Wang et al., 2024). Continuous cropping can substantially alter soil microbial communities and disrupt soil–plant–microbe interactions, contributing to continuous-cropping obstacles (Tan et al., 2021; Mao and Jiang, 2021). In pepper production systems, prolonged continuous cropping has been associated with altered microbial diversity and enrichment of soil-borne pathogenic fungi (Mao and Jiang, 2021), which may increase the risk of soil-borne diseases such as *Fusarium* wilt and constrain sustainable pepper production. Therefore, environmentally friendly agronomic strategies that improve soil ecological functions and potentially reduce the relative abundance of disease-associated microorganisms are needed.

The rhizosphere is a dynamic interface between plant roots and soil that plays central roles in nutrient transformation, microbial recruitment, and plant health. Root-derived carbon compounds and other exudates can selectively influence microbial colonization and rhizosphere microbiome assembly and functioning (Berendsen et al., 2012). The rhizosphere microbiome is increasingly recognized as an important determinant of plant performance and soil disease suppressiveness (Andargie et al., 2023; Chen et al., 2023). Continuous monocropping can modify rhizosphere conditions and microbial interactions, potentially shifting the balance between beneficial and pathogenic microorganisms. Soils with greater resistance to continuous-cropping obstacles generally exhibit higher organic matter, total nitrogen, available phosphorus, nitrate nitrogen, and bacterial diversity (Tan et al., 2021). Maintaining a diverse and functionally stable rhizosphere microbiome is therefore important for sustaining soil fertility and crop productivity under continuous cropping.

Intercropping, defined as the simultaneous cultivation of two or more crop species in the same field, is an important ecological intensification strategy for improving resource-use efficiency and agroecosystem stability. Compared with monocropping, intercropping promotes complementary utilization of light, water, nutrients, and spatial resources, thereby enhancing productivity and sustainability (Lai et al., 2022). These benefits may be particularly pronounced when legumes are included because their symbiotic associations with rhizobia enable biological nitrogen fixation and influence soil nitrogen availability and nutrient cycling. Accordingly, legume-based intercropping can affect nitrogen transfer, soil microbial communities, microbial activity, and nutrient cycling (Lai et al., 2022). For example, proso millet–mung bean intercropping increased soil nitrogen availability and altered bacterial and fungal community composition, with these changes associated with nitrogen accumulation and grain yield (Dang et al., 2020). Thus, the benefits of legume-based intercropping may extend beyond aboveground resource complementarity to important belowground processes.

Intercropping can further modify the rhizosphere microenvironment through changes in root distribution, carbon inputs, nutrient availability, and plant–microbe interactions. Melon–cowpea intercropping increased soil total nitrogen, available phosphorus, and total organic carbon while altering extracellular enzyme activities and bacterial community composition (Cuartero et al., 2022). Similarly, mung bean-based intercropping has been shown to modify rhizosphere nutrient availability, enzyme activities, microbial diversity, and microbial network structure (Liu et al., 2024; Gong et al., 2024). These findings suggest that leguminous companion crops can simultaneously influence soil nutrient cycling and rhizosphere microbial community structure.

Soil microorganisms are key regulators of soil fertility and plant productivity through their roles in organic matter decomposition, nutrient mineralization, nitrogen cycling, and disease suppression. Their composition and functional potential are strongly influenced by cropping systems and plant diversity (Andargie et al., 2023; Banerjee and van der Heijden, 2023). Intercropping may improve plant performance by regulating microorganisms involved in carbon and nutrient transformation and by promoting microbial interactions that contribute to disease suppression. Mung bean-based intercropping has been associated with changes in nitrogen fractions, nitrogen-related enzyme activities, and bacterial and fungal communities (Liu et al., 2024; Gong et al., 2024). In addition, rhizosphere microbiome diversification may enhance disease suppressiveness through competition, niche differentiation, antagonism, and parasitism (Andargie et al., 2023; Chen et al., 2023; Spooren et al., 2024). Disease-suppressive soils are shaped by interactions among soil properties, microbial communities, beneficial microorganisms, and pathogens, making rhizosphere microbiome manipulation an important strategy for sustainable disease management (Spooren et al., 2024; Sagova-Mareckova et al., 2023).

Evidence from pepper production systems further supports the potential of companion planting to alleviate continuous-cropping constraints. Companion planting can alter soil enzyme activities, bacterial and fungal communities, and microbial interaction networks in continuously cropped pepper soil (Gao and Zhang, 2023). Pepper–maize intercropping has also been reported to increase microbial diversity and reshape rhizosphere microbial and metabolite profiles, while increasing available phosphorus, available potassium, and pH and enriching potentially beneficial taxa (Chen et al., 2024). However, most studies of pepper companion planting have focused on non-leguminous crops, and the effects of legume-based intercropping on plant nutrient accumulation, rhizosphere nutrient availability, soil enzyme activities, and bacterial and fungal communities in continuously cropped pepper remain insufficiently characterized (Chen et al., 2024; Gao and Zhang, 2023). In particular, it remains unclear whether changes in these aboveground and belowground variables occur together under pepper–mung bean intercropping and how they are associated with pepper yield. Mung bean (*Vigna radiata* L.) is a short-duration legume with strong adaptability to diversified cropping systems and potential for biological nitrogen fixation. Although mung bean intercropping can modify rhizosphere microbial communities, nutrient availability, enzyme activities, and microbial networks (Dang et al., 2020; Liu et al., 2024; Gong et al., 2024), whether these changes can alleviate continuous-cropping constraints in pepper remains unclear.

We hypothesized that mung bean intercropping would improve pepper productivity by increasing rhizosphere nutrient availability and enzyme activity and by restructuring bacterial and fungal communities. To test these hypotheses, a field experiment was conducted in a long-term continuously cropped pepper field to evaluate pepper growth and yield, plant nutrient accumulation, rhizosphere soil properties, enzyme activities, and bacterial and fungal communities using agronomic measurements, soil biochemical analyses, and high-throughput amplicon sequencing. This study provides insights into belowground ecological processes associated with legume-based intercropping and pepper productivity under continuous-cropping conditions.

## 2 Materials and methods

### 2.1 Experimental site and plant materials

The field experiment was conducted from May to October 2024 at the experimental station of the Dezhou Academy of Agricultural Sciences, Shandong Province, China (116°45′E, 37°24′N). The field had been continuously cropped with pepper for five consecutive years before the experiment. The soil was classified as loam, the initial soil properties were as follows: organic matter, 13.52 g kg^-^¹; organic carbon, 7.84 g kg^-^¹; total nitrogen, 0.81 g kg^-^¹; available nitrogen, 44.09 mg kg^-^¹; total phosphorus, 0.95 g kg^-^¹; available phosphorus, 36.45 mg kg^-^¹; total potassium, 20.03 g kg^-^¹; available potassium, 152.00 mg kg^-^¹; and pH, 8.49. The pepper cultivar used in the experiment was the clustered chili pepper cultivar ‘Bolatiangjiao 60’, obtained from Hunan Xingshu Seed Industry Co., Ltd., China. The mung bean cultivar ‘Delü 0518’, characterized by a compact dwarf plant type, was provided by the Institute of Minor Grain Crops, Dezhou Academy of Agricultural Sciences.

### 2.2 Experimental design and field management

The experiment was arranged as a randomized complete block design with five blocks. Each block contained the two cropping treatments, MP and IP, resulting in 10 experimental plots in total. Each treatment was represented by five independent plots, and each plot was considered an independent experimental unit for statistical analysis. Each plot was 28.8 m² in area and 6 m in length. In MP, pepper was planted in eight rows using a wide–narrow row arrangement. The spacing between wide rows was 80 cm, the spacing between narrow rows was 60 cm, and the within-row plant spacing was 35 cm. In IP, pepper and mung bean were arranged at a 2:1 row ratio, with one mung bean row planted in the middle of the wide pepper row. The distance between the mung bean row and each adjacent pepper row was 40 cm, and the within-row plant spacing of mung bean was 20 cm. Pepper planting density was kept consistent between treatments to minimize potential effects of differences in pepper population density on plant growth and yield. Pepper seedlings were raised in plug trays beginning on March 20, 2024, and transplanted into the field on May 9, 2024. Mung bean was directly sown in the field on May 11, 2024, two days after pepper transplantation. Except for the planting pattern, all other field management practices, including fertilization, irrigation, and pest management, were kept consistent between treatments and followed local conventional production practices.

### 2.3 Determination of plant growth characteristics and yield

After establishment, pepper plants with uniform growth were randomly selected and permanently marked in each plot. Plant height and stem diameter were measured at four developmental stages: seedling, peak flowering, peak fruiting, and harvest. For biomass determination, three representative pepper plants were collected from each plot at each developmental stage. Plants were separated into roots, stems, leaves, and fruits. Samples were oven-dried at 75 °C to constant weight and then weighed to determine dry biomass. At the harvest stage, three representative pepper plants were randomly selected from each plot to determine yield-related traits, including fruit number per plant and average fruit weight. Pepper yield was calculated based on the cumulative fruit weight harvested from each plot and expressed on a unit-area basis.

### 2.4 Determination of nitrogen, phosphorus, and potassium concentrations in plant tissues

Plant tissues used for N, P, and K analysis were collected at harvest stage from the roots, stems, leaves, and fruits of pepper plants sampled from each plot. The samples were oven-dried at 75 °C to constant weight and finely ground before nutrient analysis. Approximately 0.25 g of each sample was digested with concentrated H_2_SO_4_ and H_2_O_2_ until a clear digest was obtained according to established procedures (Thomas et al., 1967). The digests were then diluted to a fixed volume with deionized water. Total nitrogen (N) concentration was determined using a continuous-flow analyzer (AA3, SEAL Analytical, Norderstedt, Germany) following acid digestion based on automated colorimetric analysis (Varley, 1966). Total phosphorus (P) concentration was determined using the molybdenum–antimony–ascorbic acid colorimetric method (Murphy and Riley, 1962). Total potassium (K) concentration was determined by flame photometry following H_2_SO_4_–H_2_O_2_ digestion (Havre, 1961). Nutrient concentrations were expressed on a dry-weight basis.

### 2.5 Determination of rhizosphere soil nutrients and enzyme activities

Rhizosphere soil samples were collected at the pepper harvest stage. At pepper harvest, five pepper plants with uniform growth were randomly selected from each plot. Rhizosphere soil was collected from the five plants using the shaking-off method and pooled within each plot to obtain one composite rhizosphere soil sample per plot. Thus, each plot contributed one composite sample, and the five plot-level samples per treatment were treated as five independent biological replicates for soil and rhizosphere microbial analyses. Soil samples intended for nutrient analysis were air-dried, gently crushed, and passed through a 2-mm sieve. Samples intended for enzyme activity assays were kept fresh at 4 °C and analyzed as soon as possible. Soil total nitrogen (TN) was determined using the semi-micro Kjeldahl method after digestion with concentrated H_2_SO_4_ (Nelson and Sommers, 1980). Soil total phosphorus (TP) was determined using the NaOH fusion–molybdenum–antimony colorimetric method, and total potassium (TK) was determined using the NaOH fusion–flame photometric method, following Bao (Bao, 2000). Soil alkali-hydrolyzable nitrogen (AN), representing a readily mineralizable nitrogen fraction, was determined using the alkaline hydrolysis–diffusion method. Soil samples were treated with an alkaline solution to release NH_3_, which was absorbed by boric acid and subsequently quantified by titration with standard acid (Zhu et al., 2023). Available phosphorus (AP) was extracted with 0.5 M NaHCO_3_ (pH 8.5) according to the Olsen method, and phosphorus concentration in the extract was determined using the molybdenum blue method (Olsen and Sommers, 1983). Available potassium (AK) was extracted with 1.0 M neutral ammonium acetate (NH_4_OAc, pH 7.0), and K concentration in the extract was determined by flame photometry (Peng et al., 2022). Soil urease activity was determined by measuring the amount of NH_4_^+^ released during urea hydrolysis according to Tabatabai and Bremner (Tabatabai and Bremner, 1972). Soil dehydrogenase activity was determined using the triphenyltetrazolium chloride (TTC) reduction method (Małachowska-Jutsz and Matyja, 2019). Soil catalase activity was determined by measuring H_2_O_2_ decomposition using the KMnO_4_ titration method (Johnson and Temple, 1964).

### 2.6 Rhizosphere microbial community analysis

Rhizosphere soil samples stored at −80 °C were used for microbial community analysis. DNA extraction, PCR amplification, library preparation, and sequencing were performed by Shanghai Majorbio Bio-pharm Technology Co., Ltd., China. The bacterial 16S rRNA gene was amplified using the primers 338F (5′-ACTCCTACGGGAGGCAGCA-3′) and 806R (5′-GGACTACHVGGGTWTCTAAT-3′). The fungal internal transcribed spacer (ITS) region was amplified using ITS1F (5′-CTTGGTCATTTAGAGGAAGTAA-3′) and ITS2 (5′-GCTGCGTTCTTCATCGATGC-3′). PCR products were purified, quantified, and pooled in equimolar proportions before high-throughput sequencing. Raw sequencing data were processed using the bioinformatic pipeline provided by the sequencing facility. Amplicon sequence variants (ASVs) were taxonomically annotated, and microbial community diversity and composition were subsequently analyzed. The same processing criteria were applied to all samples.

### 2.7 Statistical analysis

All statistical analyses were performed using the Majorbio Cloud Platform. Differences between MP and IP were assessed using Student’s *t*-test when the assumptions of parametric analysis were satisfied; otherwise, the Wilcoxon rank-sum test was used. Statistical significance was defined as *P* < 0.05. Alpha diversity indices, including Ace, Chao, Shannon, and Simpson indices, were calculated for bacterial and fungal communities. Principal coordinate analysis (PCoA) was conducted based on Bray–Curtis dissimilarities to visualize differences in bacterial and fungal community composition between MP and IP. Differences in community composition were statistically tested using permutational multivariate analysis of variance (PERMANOVA) based on the Bray–Curtis dissimilarity matrices. The R² and P values were reported for both bacterial and fungal communities. Differentially abundant microbial taxa were identified using the Wilcoxon rank-sum test with *P* < 0.05 as the significance threshold. Spearman correlation analysis was used to evaluate associations between dominant microbial taxa, rhizosphere soil properties, enzyme activities, and pepper productivity-related traits. Figures were generated using GraphPad Prism and/or the Majorbio analysis platform.

## 3 Results

### 3.1 IP promoted pepper yield formation

To evaluate the effects of IP on pepper growth and productivity, several agronomic and yield-related traits were measured at the harvest stage. Compared with MP, IP did not significantly affect plant height, stem diameter, or fruit number per plant (**Figures 1A–C**; **Supplementary Table S1**). In contrast, average fruit weight was significantly higher under IP than under MP (**Figure 1D**). Consistent with this response, IP significantly increased pepper yield per unit area (**Figure 1E**). These results indicated that the productivity advantage associated with IP may be primarily reflected in fruit development and yield formation rather than in the measured vegetative growth traits.

**Figure 1:**
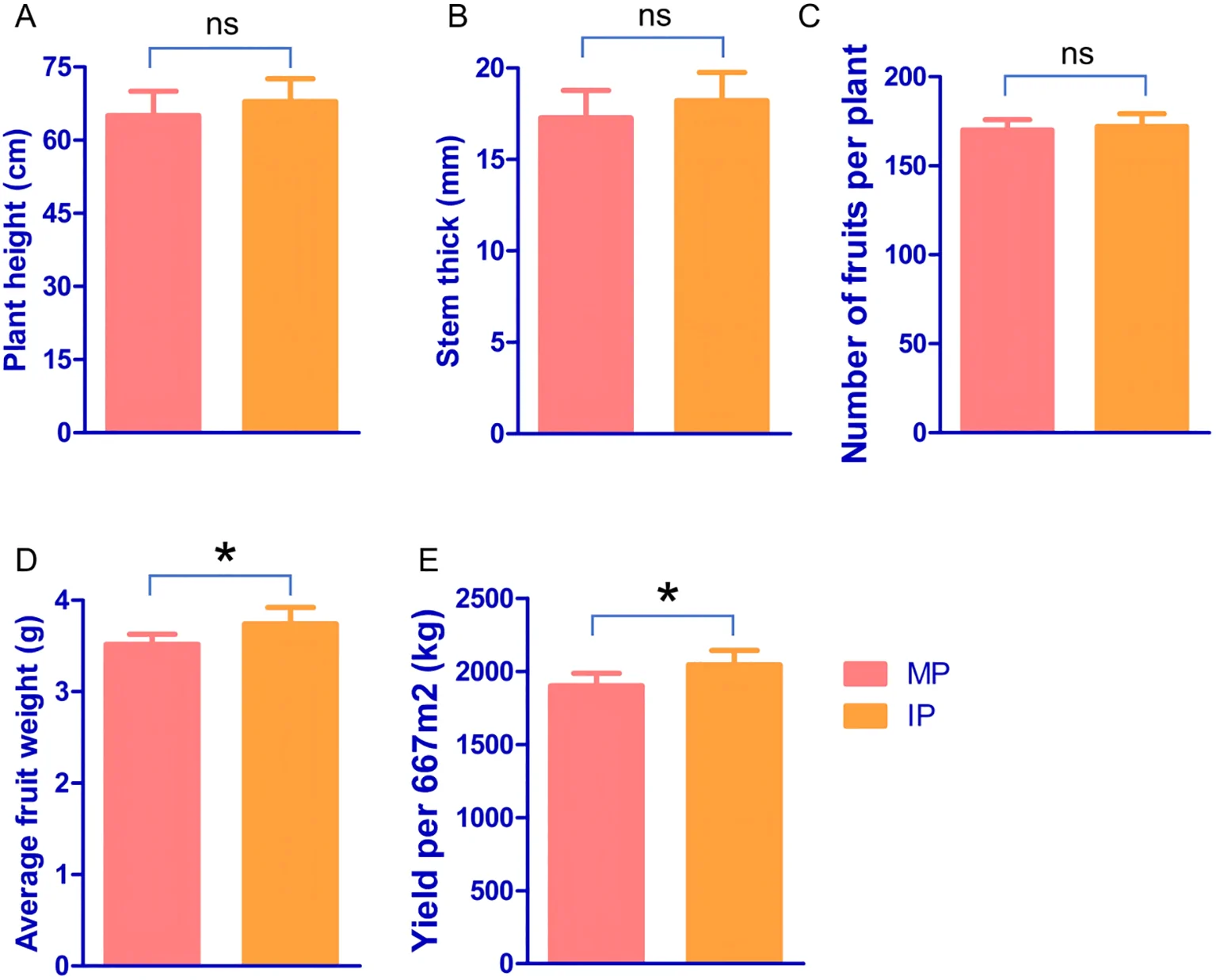
Effects of IP on pepper growth and yield formation at the harvest stage. **(A)** Plant height; **(B)** stem diameter; **(C)** fruit number per plant; **(D)** average fruit weight; and **(E)** pepper yield under MP and IP. Data are presented as means ± standard deviation (SD) of five biological replicates. Asterisks indicate significant differences between treatments (*P* < 0.05), whereas ns indicates no significant difference (*P* ≥ 0.05), according to Student’s *t*-test.

### 3.2 IP altered nutrient accumulation patterns in different pepper organs

To determine whether IP affected nutrient accumulation in pepper, N, P, and K accumulation was analyzed separately in roots, stems, leaves, and fruits (**Figure 2**). Nitrogen accumulation showed organ-specific responses to IP. Compared with MP, IP significantly increased N accumulation in roots and fruits, whereas no significant differences were detected in stems or leaves (**Figure 2A**). Fruits represented the major N accumulation compartment among the organs examined. Phosphorus accumulation was also affected by IP, with significant increases observed particularly in roots and fruits (**Figure 2B**). Similarly, compared with MP, IP significantly increased K accumulation in roots, stems, and fruits (**Figure 2C**). Collectively, these results demonstrated that IP may modify the accumulation and distribution of N, P, and K among pepper organs, with reproductive tissues showing pronounced responses.

**Figure 2:**
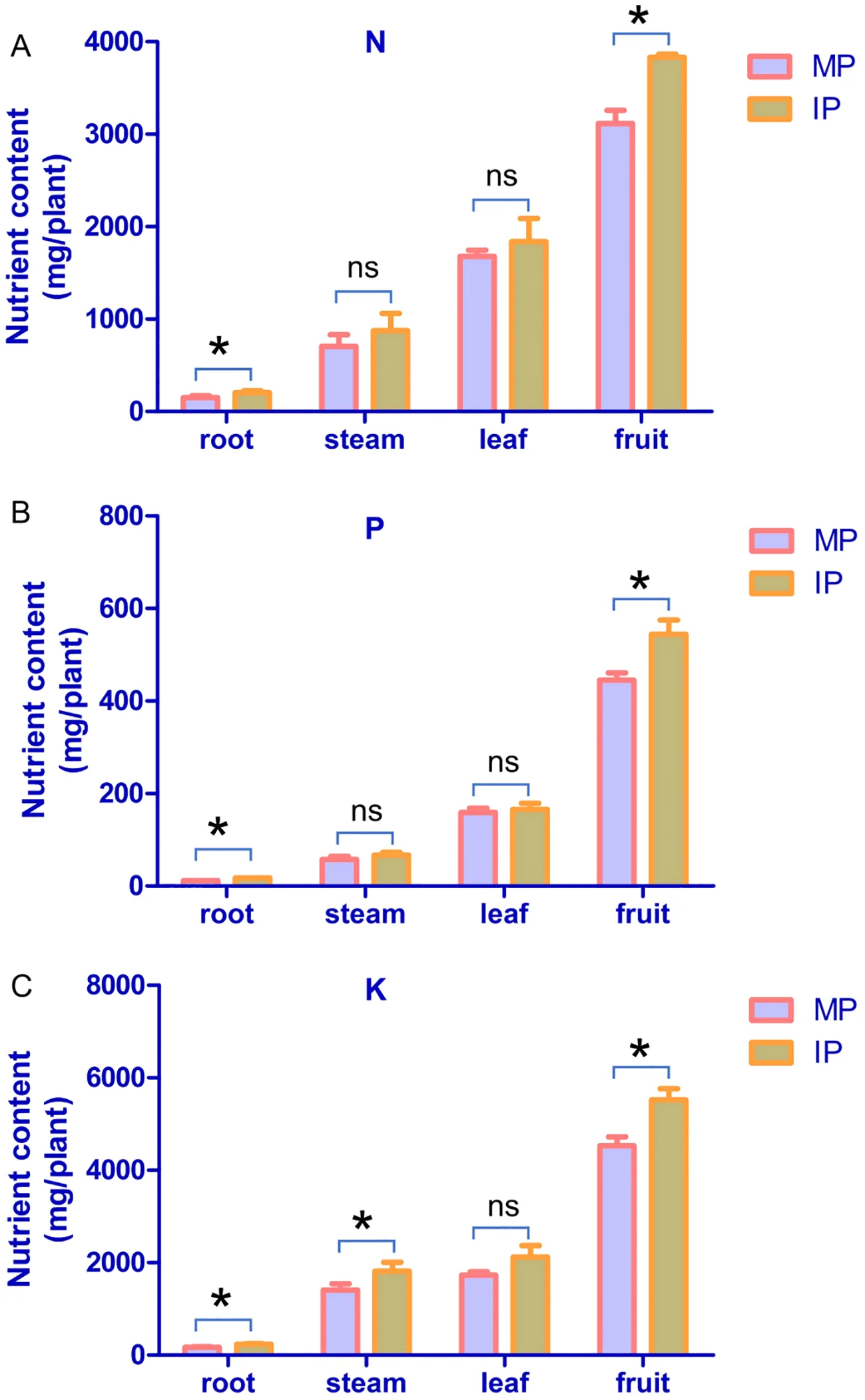
Effects of IP on nitrogen, phosphorus, and potassium accumulation in pepper organs. **(A)** Nitrogen accumulation; **(B)** phosphorus accumulation; and **(C)** potassium accumulation in roots, stems, leaves, and fruits of pepper under MP and IP. Data are presented as means ± SD of five biological replicates. Asterisks indicate significant differences between treatments (P < 0.05), whereas ns indicates no significant difference (P ≥ 0.05), according to Student’s t-test.

### 3.3 IP increased rhizosphere nutrient availability and soil enzyme activities

To determine whether IP modified rhizosphere soil fertility and biological activity, soil nutrient concentrations and enzyme activities were measured (**Figure 3**). Compared with MP, IP significantly increased the availability of N, P, and K in the pepper rhizosphere (**Figures 3A–C**), whereas the concentrations of soil TN, TP, and TK did not differ significantly between treatments (**Supplementary Figure S1**). These results showed that IP affected available nutrient concentrations but not total soil N, P, and K concentrations. IP also significantly increased soil dehydrogenase, urease, and catalase activities compared with MP (**Figures 3D–F**). The increases in enzyme activities, together with the higher concentrations of available nutrients, indicated that IP may be associated with changes in rhizosphere biochemical processes and nutrient availability.

**Figure 3:**
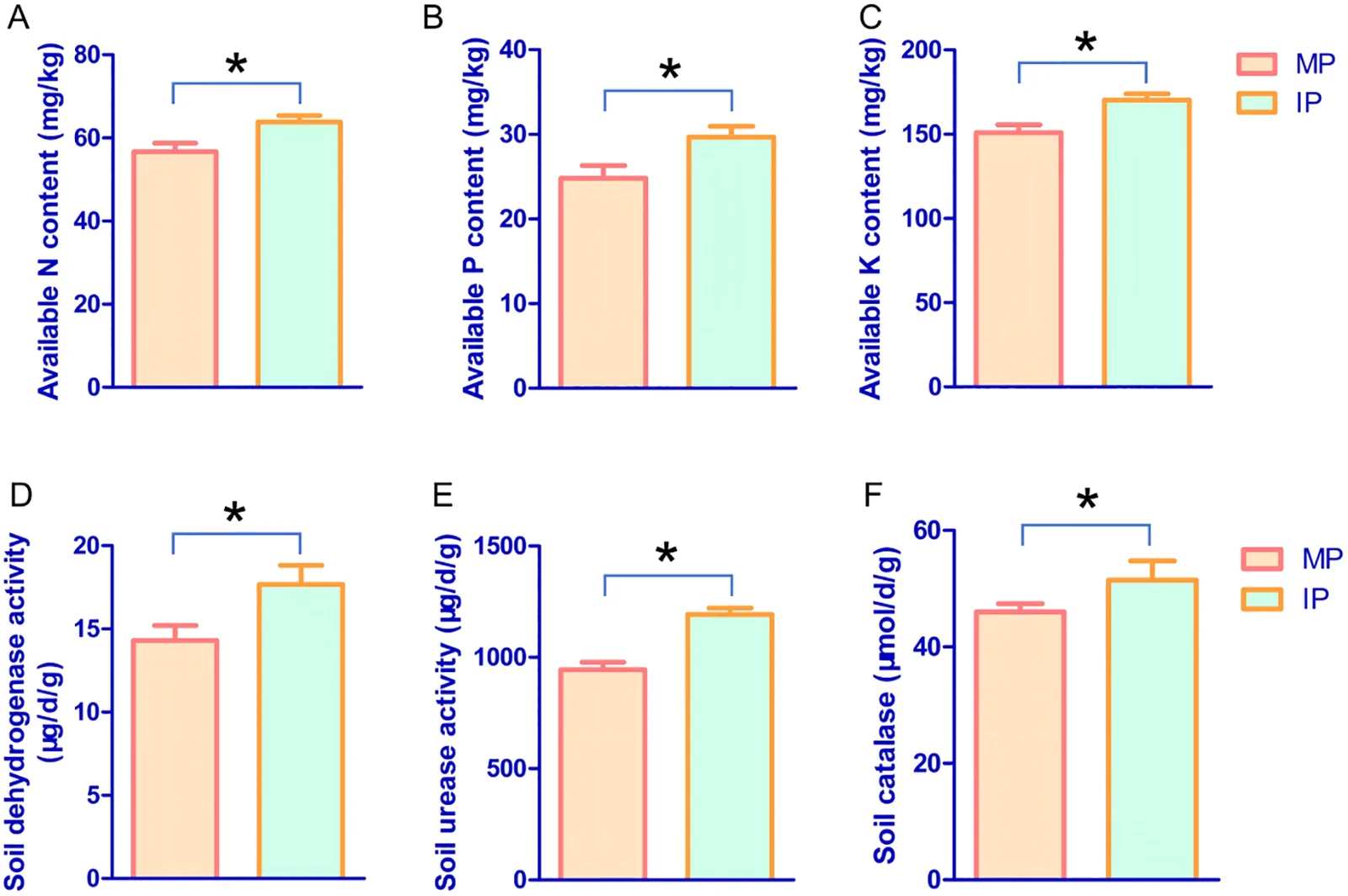
Effects of IP on rhizosphere soil nutrient availability and enzyme activities. **(A)** Available nitrogen; **(B)** available phosphorus; **(C)** available potassium; **(D)** urease activity; **(E)** dehydrogenase activity; and **(F)** catalase activity in rhizosphere soil under MP and IP. Data are presented as means ± SD of five biological replicates. Asterisks indicate significant differences between treatments (*P* < 0.05), according to Student’s *t*-test.

### 3.4 IP reshaped rhizosphere fungal community composition

High-throughput sequencing was performed to characterize fungal communities in the pepper rhizosphere under MP and IP (**Supplementary Table S2**). IP significantly altered fungal richness, as indicated by differences in the Ace and Chao indices, whereas the Shannon and Simpson indices did not differ between treatments (**Figures 4A–D**). Bray–Curtis-based PCoA showed separation between MP and IP fungal communities (**Figure 4E**), PERMANOVA also indicated a significant difference in fungal community composition between MP and IP (*R*² = 0.676, *P* = 0.005), providing a statistical test of the treatment-associated difference in fungal community composition. At the genus level, *Mortierella*, *Botryotrichum*, *Bisifusarium*, *Pseudombrophila*, *Gibellulopsis*, *Neocosmospora*, and *Conocybe* were among the dominant genera in both treatments (**Figure 4F**; **Supplementary Figure S2**). Wilcoxon rank-sum analysis identified significant differences in several fungal genera between treatments (**Figure 4G**), with *Bisifusarium* and *Neocosmospora* enriched under MP and *Conocybe* enriched under IP.

**Figure 4:**
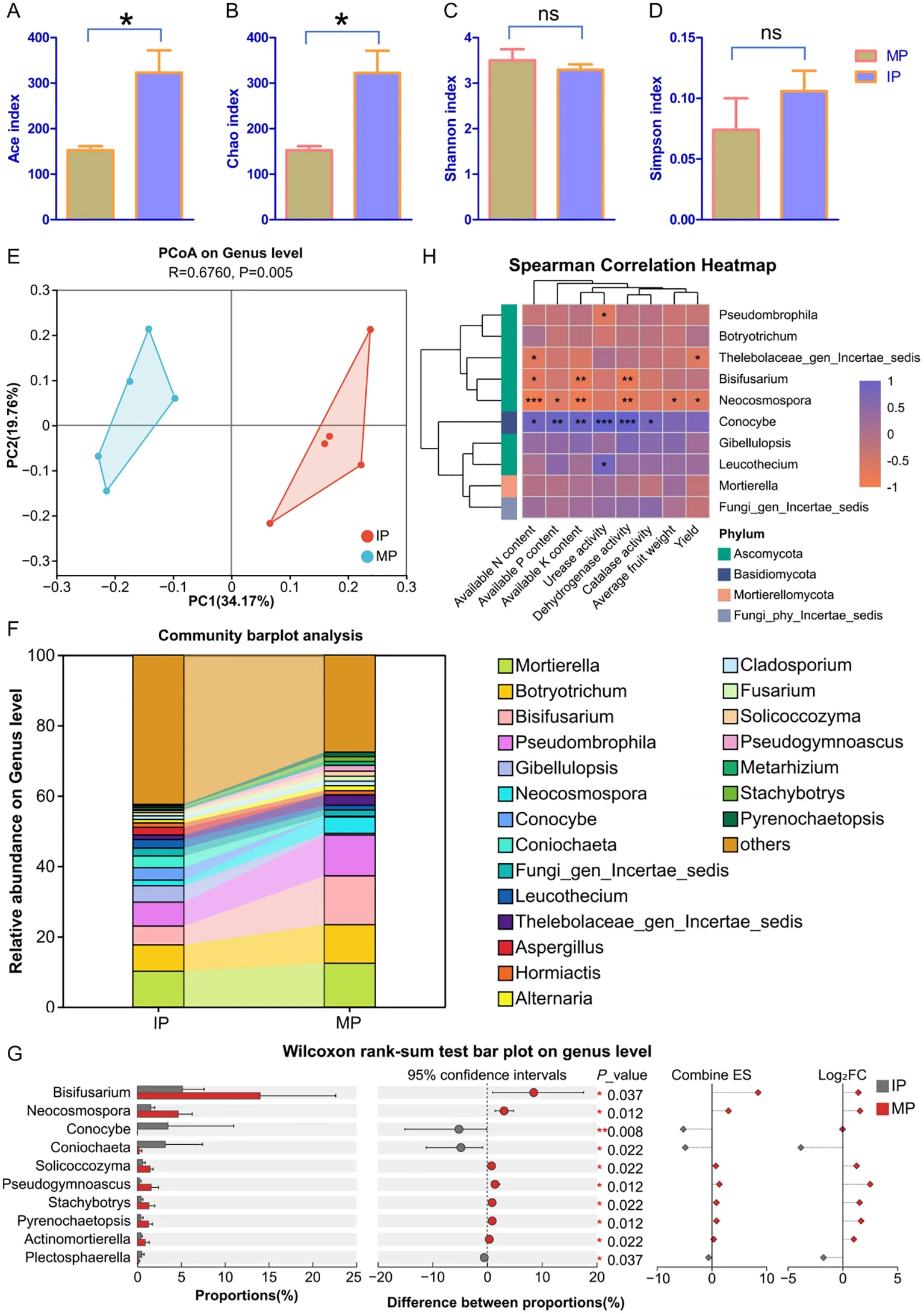
Effects of IP on rhizosphere fungal community diversity and composition. **(A–D)** Alpha diversity indices of fungal communities, including Ace, Chao, Shannon, and Simpson indices, respectively. Data are presented as means ± SD of five biological replicates. Asterisks indicate significant differences between treatments (*P* < 0.05), whereas ns indicates no significant difference (*P* ≥ 0.05), according to Student’s *t*-test. **(E)** PCoA based on Bray–Curtis dissimilarities showing differences in fungal community composition between MP and IP. PERMANOVA based on Bray–Curtis dissimilarities: *R*² = 0.676, *P* = 0.005. **(F)** Relative abundance of dominant fungal genera. **(G)** Differentially abundant fungal genera between MP and IP identified using the Wilcoxon rank-sum test (*P* < 0.05). **(H)** Spearman correlation analysis between dominant fungal taxa, rhizosphere soil properties, and pepper productivity-related traits. The color gradient represents the strength and direction of the correlations.

Spearman correlation analysis revealed associations between dominant fungal taxa, rhizosphere soil properties, and pepper productivity (**Figure 4H**). *Conocybe* was positively correlated with available N, P, and K, urease, dehydrogenase, and catalase activities, as well as average fruit weight and pepper yield. In contrast, *Neocosmospora* and *Bisifusarium* showed predominantly negative correlations with soil nutrient and biochemical variables and yield-related traits. Overall, IP was associated with distinct rhizosphere fungal community composition and changes in the relative abundances of several fungal taxa correlated with soil properties and pepper productivity.

### 3.5 IP altered rhizosphere bacterial diversity and community structure

High-throughput sequencing was performed to characterize bacterial communities in the pepper rhizosphere under MP and IP (**Supplementary Table S3**). Alpha diversity indices, including Ace, Chao, Shannon, and Simpson, differed significantly between treatments (**Figures 5A–D**), indicating changes in bacterial richness and diversity under IP. Bray–Curtis-based PCoA showed separation between MP and IP bacterial communities (**Figure 5E**). PERMANOVA indicated a significant difference in bacterial community composition between MP and IP (*R*² = 0.928, P = 0.01) for the treatment-associated difference in bacterial community composition. The dominant bacterial taxa included *Vicinamibacterales*, *Vicinamibacteraceae*, *Gemmatimonadaceae*, *Pyrinomonadaceae*, *bacteriap25*, *Geminicoccaceae*, *KD4-96*, *Actinomarinales*, and *Rokubacteriales* (**Figure 5F**; **Supplementary Figure S3**). Compared with MP, IP increased the relative abundances of *Vicinamibacterales*, *Vicinamibacteraceae*, *KD4-96*, and *Rokubacteriales*, whereas *bacteriap25*, *Nitrospira*, and *S0134_terrestrial_group* were more abundant under MP. Differential abundance analysis identified seven taxa enriched under IP, with *Vicinamibacterales* showing the strongest treatment difference (**Figure 5G**). In contrast, *bacteriap25*, *Nitrospira*, and *S0134_terrestrial_group* were significantly enriched under MP.

**Figure 5:**
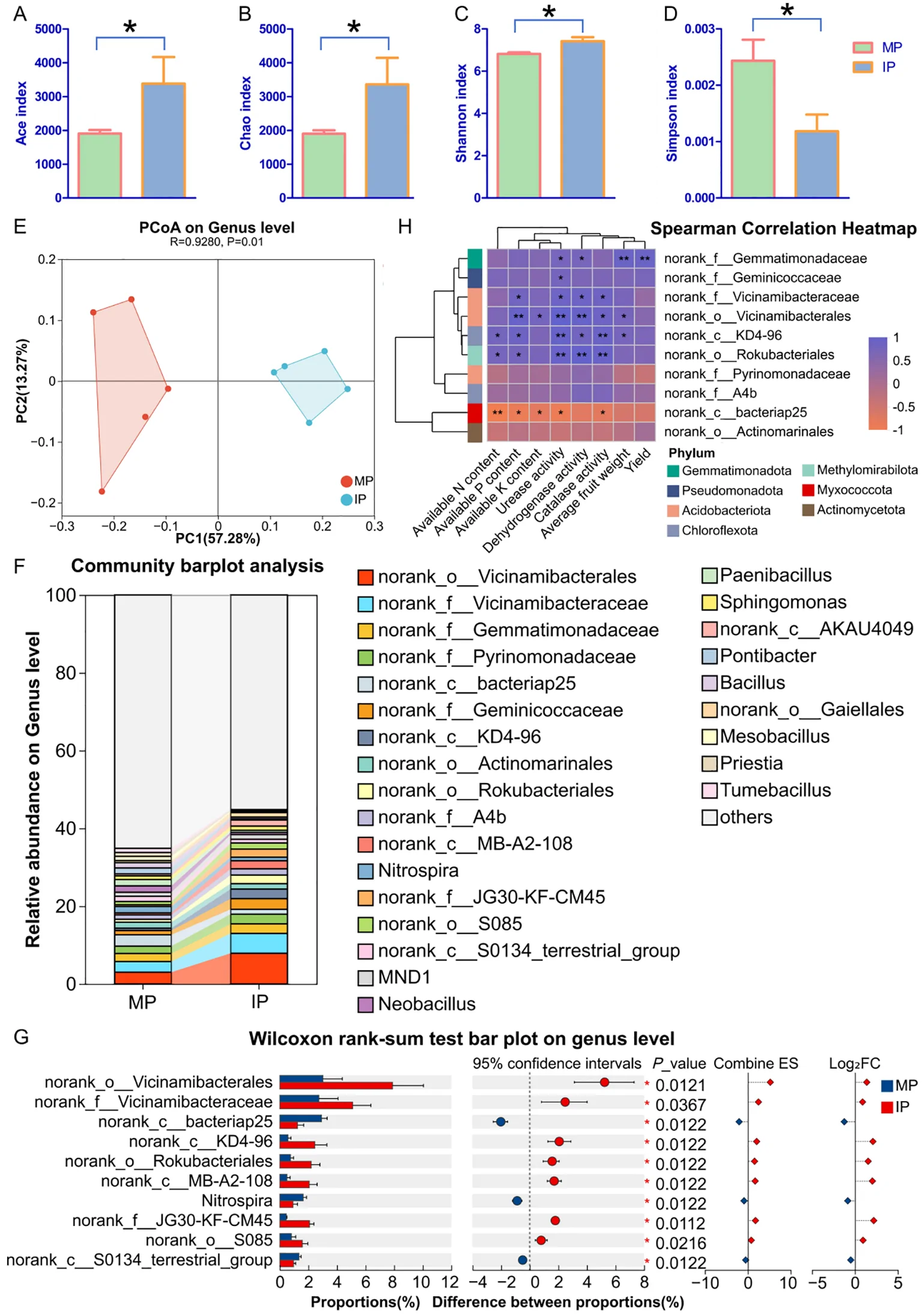
Effects of IP on rhizosphere bacterial community diversity and composition. **(A–D)** Alpha diversity indices of bacterial communities, including Ace, Chao, Shannon, and Simpson indices, respectively. Data are presented as means ± SD of five biological replicates. Asterisks indicate significant differences between treatments (*P* < 0.05), whereas ns indicates no significant difference (*P* ≥ 0.05), according to Student’s *t*-test. **(E)** PCoA based on Bray–Curtis dissimilarities showing differences in bacterial community composition between MP and IP. PERMANOVA based on Bray–Curtis dissimilarities: *R*² = 0.928, *P* = 0.01. **(F)** Relative abundance of dominant bacterial taxa. **(G)** Differentially abundant bacterial taxa between MP and IP identified using the Wilcoxon rank-sum test (*P* < 0.05). **(H)** Spearman correlation analysis between dominant bacterial taxa, rhizosphere soil properties, and pepper productivity-related traits. The color gradient represents the strength and direction of the correlations.

Spearman correlation analysis showed that IP-enriched taxa, particularly *Vicinamibacterales*, *Vicinamibacteraceae*, *KD4-96*, and *Rokubacteriales*, were positively correlated with rhizosphere nutrient availability, soil enzyme activities, average fruit weight, and pepper yield (**Figure 5H**). Conversely, *bacteriap25*, which was enriched under MP, showed predominantly negative correlations with the measured soil nutrient and enzyme variables. Overall, IP was associated with marked shifts in bacterial community composition and its relationships with rhizosphere soil properties and pepper productivity.

## 4 Discussion

### 4.1 IP improved pepper productivity through enhanced nutrient acquisition

Continuous monocropping can impair sustainable vegetable production by inducing soil nutrient imbalance, microbial community restructuring, and accumulation of soil-borne pathogens (Tan et al., 2021; Chen et al., 2022). In the present study, IP significantly increased average fruit weight and final yield, whereas plant height, stem diameter, and fruit number per plant were unaffected (**Figure 1**). This result suggests that the yield advantage of IP was primarily associated with fruit development rather than enhanced vegetative growth. Intercropping can improve productivity through complementary use of light, water, nutrients, and spatial resources (Lai et al., 2022; Cappelli et al., 2022), while legume inclusion may additionally modify nutrient cycling and plant–microbe interactions (Lai et al., 2022; Chamkhi et al., 2022; Brooker et al., 2015). Consistent with these effects, IP increased N, P, and K accumulation in pepper, particularly in roots and fruits (**Figure 2**). Increased nutrient accumulation in reproductive tissues may be associated with greater fruit weight and yield and may partly explain the yield response observed under IP. This pattern is consistent with the possibility that improved nutrient acquisition and allocation supported fruit development under intercropping. However, the present study does not establish that increased nutrient accumulation directly caused the increase in fruit weight or yield. However, biological N fixation and direct N transfer from mung bean to pepper were not quantified. Thus, increased pepper N accumulation should not be interpreted as evidence of direct N transfer. Instead, mung bean may have indirectly modified the rhizosphere through changes in nutrient competition, root activity, organic matter inputs, and microbial processes, consistent with plant–soil–microbe feedbacks promoted by plant diversification (Cappelli et al., 2022; Chamkhi et al., 2022). Overall, IP may improve pepper productivity by enhancing nutrient acquisition and allocation, particularly to reproductive tissues, rather than by promoting general vegetative growth.

### 4.2 IP was associated with increased rhizosphere nutrient availability and soil enzyme activities

IP significantly increased available N, P, and K in the pepper rhizosphere, whereas total soil N, P, and K remained unchanged (**Figure 3**; **Supplementary Figure S1**). This suggests that IP primarily affected readily available nutrient pools rather than total nutrient stocks during the experimental period. IP also increased urease, dehydrogenase, and catalase activities, which are commonly associated with soil N transformation, microbial oxidative activity, and oxidative processes, respectively (Tabatabai and Bremner, 1972; Małachowska-Jutsz and Matyja, 2019; Johnson and Temple, 1964). These results show that IP was associated with higher enzyme activities at pepper harvest. The increased nutrient availability may reflect interacting effects of mung bean roots, carbon inputs, microbial turnover, nutrient competition, and nutrient transformation rather than root exudation alone. Because soil properties and enzyme activities were measured at a single time point, these data cannot determine when the differences emerged or whether they persisted throughout the growing season. Previous studies have similarly shown that intercropping can increase soil nutrient availability and enzyme activity (Cuartero et al., 2022; Curtright and Tiemann, 2021). Mung bean-based systems can also modify soil N fractions, N-related enzyme activities, and microbial communities (Liu et al., 2024; Gong et al., 2024). Therefore, IP may improve the rhizosphere biochemical environment and nutrient availability. Because enzyme activities were measured at a single stage, however, the persistence of these effects throughout the growing season remains to be determined.

### 4.3 IP reshaped fungal communities and reduced potentially pathogen-associated taxa

IP substantially altered rhizosphere fungal community structure. Continuous cropping can generate crop-specific soil legacies through repeated root-derived inputs, changes in soil properties, and selection of host-associated microorganisms (Tan et al., 2021; Chen et al., 2022). Long-term pepper monocropping has been associated with reduced microbial diversity and enrichment of *Fusarium*- and *Gibberella*-related taxa (Mao and Jiang, 2021). In the present study, *Bisifusarium* and *Neocosmospora* were significantly more abundant under MP than IP (**Figure 4**). These genera contain species associated with plants and, in some cases, plant diseases (Kamali-Sarvestani et al., 2022; Wang et al., 2022). Their lower relative abundance under IP may therefore reflect a shift in the fungal community associated with continuous cropping. One possible explanation is that mung bean intercropping altered rhizosphere resource availability and root-derived inputs, thereby changing the ecological conditions for fungal colonization and competition. Changes in nutrient availability and soil biochemical conditions may have indirectly influenced the relative representation of different fungal taxa. However, genus-level relative abundance cannot confirm pathogen activity, and pathogen density, pathogenicity, and disease incidence were not measured. Thus, these results should be interpreted as reduced relative abundances of potentially pathogen-associated taxa, specifically *Bisifusarium* and *Neocosmospora*, rather than evidence of reduced relative abundance of potentially pathogen-associated taxa or disease control. Pathogen abundance, pathogenicity, and disease incidence were not measured in this study. This pattern is consistent with previous studies showing that crop diversification can restructure microbial communities in continuous-cropping systems (Gao and Zhang, 2023; Gao et al., 2022), while green manure intercropping has been associated with soil microbiome reconstruction and reduced Fusarium wilt severity (Yang et al., 2022). IP also significantly increased *Conocybe*, a genus commonly detected in soil and organic substrates and generally considered saprotrophic. Its enrichment may reflect altered substrate availability and fungal resource use under IP. Changes in root-derived inputs and organic substrates may have modified the resources available to soil fungi, potentially contributing to the observed shift in fungal community composition. However, amplicon sequencing cannot establish its actual decomposition activity or contribution to nutrient mineralization. Positive correlations between *Conocybe* and available N, P, and K, soil enzyme activities, average fruit weight, and yield suggest that its abundance was associated with a more favorable rhizosphere environment. This association may reflect a shared response of fungal community composition and pepper productivity to changes in soil resource availability, rather than a direct effect of *Conocybe* on nutrient cycling or yield. Nevertheless, the specific ecological functions underlying these relationships remain to be verified. Overall, IP may shift the fungal community away from greater representation of potentially pathogen-associated fusarioid taxa and toward a community containing more taxa such as *Conocybe*.

### 4.4 IP reshaped rhizosphere bacterial communities in association with nutrient availability

IP also substantially modified bacterial community composition. *Vicinamibacterales*, *Vicinamibacteraceae*, *KD4-96*, and *Rokubacteriales* were enriched under IP, whereas *bacteriap25*, *Nitrospira*, and *S0134_terrestrial_group* were relatively more abundant under MP (**Figure 5**). Several IP-enriched taxa, particularly *Vicinamibacterales*, showed positive associations with available N, P, and K and with urease, dehydrogenase, and catalase activities. These relationships suggest that intercropping-associated changes in nutrient availability and soil biochemical conditions may have contributed to the observed bacterial community shifts. Conversely, changes in bacterial community composition may also be associated with differences in nutrient transformation processes. For example, urease activity is related to urea hydrolysis and N availability, whereas microbial processes involved in P mobilization may influence available P pools. However, because bacterial functions and functional genes were not characterized, the specific contributions of the enriched taxa to these processes remain uncertain. Correlation analysis cannot determine whether these taxa directly contributed to nutrient cycling or primarily responded to altered resource availability.

Plant diversification can restructure rhizosphere bacteria through changes in root-derived resources, nutrient availability, and plant–soil feedbacks (Cappelli et al., 2022). Consistent with this concept, companion planting and pepper–maize intercropping have been reported to alter bacterial communities in pepper soils in association with changes in soil properties (Gao and Zhang, 2023; Chen et al., 2024). The positive association between *Vicinamibacterales* and available P is particularly noteworthy because soil microbiomes possess diverse strategies for P acquisition (Wu et al., 2022). Intercropping-related changes in root-derived inputs and nutrient availability may have altered the conditions influencing microbial P acquisition and turnover. The association between *Vicinamibacterales* and available P may therefore reflect its response to changes in the rhizosphere environment or a potential link with P-related microbial processes. However, functional genes and metabolic pathways were not characterized here, and therefore the specific role of Vicinamibacterales in P acquisition cannot be established. Its simultaneous associations with multiple nutrients and enzyme activities further suggest a broader response to the altered rhizosphere environment rather than a demonstrated, P-specific function. Recent studies of mung bean-based intercropping likewise demonstrate changes in soil N fractions, enzyme activities, microbial communities, and microbial networks (Liu et al., 2024; Gong et al., 2024). Thus, bacterial community restructuring under IP may reflect responses to altered nutrient availability and soil enzyme activities. However, the present correlations do not determine whether the enriched taxa contributed to nutrient transformations or instead responded to the changed rhizosphere environment.

### 4.5 Integrated interpretation and limitations

Collectively, IP increased available N, P, and K without significantly changing total soil nutrient stocks, enhanced urease, dehydrogenase, and catalase activities, and reshaped both fungal and bacterial communities. The fungal response was characterized by reduced relative abundances of *Bisifusarium* and *Neocosmospora* and increased *Conocybe*, whereas bacterial changes included enrichment of *Vicinamibacterales*, *Vicinamibacteraceae*, *KD4-96*, and *Rokubacteriales*. Several microbial taxa were positively associated with nutrient availability, enzyme activities, fruit weight, and yield. These coordinated responses suggest that mung bean intercropping was associated with changes in the rhizosphere environment that coincided with improved pepper productivity. The increased availability of N, P, and K and the enhanced soil enzyme activities may reflect changes in nutrient transformation processes and microbial resource conditions under IP. These changes occurred alongside shifts in fungal and bacterial community composition, suggesting that nutrient availability, soil biochemical conditions, and microbial community structure were interconnected. The correlations among microbial taxa, soil properties, and yield-related traits further support these associations. However, the present study cannot determine the direction of these relationships or establish that particular taxa caused the agronomic responses.

Several limitations should be considered. First, biological N fixation and direct N transfer from mung bean to pepper were not quantified; therefore, increased pepper N accumulation cannot be attributed directly to mung bean N fixation. Second, pathogen abundance and disease incidence were not measured, precluding direct conclusions regarding disease suppression. Third, amplicon sequencing provides relative abundance rather than direct evidence of microbial activity or function. Finally, correlations among microbial taxa, soil properties, and plant productivity do not establish causal relationships. Future studies combining quantitative pathogen assays, metagenomics or metatranscriptomics, metabolomics, and microbial isolation or inoculation experiments will be valuable for clarifying how mung bean modifies the pepper rhizosphere and whether specific microorganisms directly contribute to nutrient cycling, reduced relative abundance of potentially pathogen-associated taxa, or yield improvement.

## 5 Conclusion

IP improved pepper productivity under continuous cropping, as reflected by increased average fruit weight and yield, and was associated with altered N, P, and K accumulation in pepper. IP also increased rhizosphere nutrient availability and soil enzyme activities and substantially reshaped bacterial and fungal community composition. The reduced relative abundances of *Bisifusarium* and *Neocosmospora* and the enrichment of several bacterial taxa were associated with improved rhizosphere properties and pepper productivity. Overall, these findings indicate that mung bean intercropping was associated with improved pepper productivity, increased rhizosphere nutrient availability and soil enzyme activities, and changes in bacterial and fungal community composition under continuous cropping. Because rhizosphere sampling was conducted only at pepper harvest and the microbial analyses were based on community composition and correlations, further temporal, functional, and pathogen-focused studies are needed to determine the persistence and mechanisms of these associations. Further studies integrating functional analyses and pathogen quantification are needed to clarify the mechanisms underlying these associations.

## Data Availability

The raw amplicon sequencing data generated in this study have been deposited in the NCBI Sequence Read Archive (SRA) under BioProject accession PRJNA1529062.

## References

[B1] Andargie Y. E. Lee G. Jeong M. Tagele S. B. Shin J.-H. (2023). Deciphering key factors in pathogen-suppressive microbiome assembly in the rhizosphere. Front. Plant Sci. 14, 1301698. doi: 10.3389/fpls.2023.1301698 38116158 PMC10728675

[B2] Banerjee S. van der Heijden M. G. A. (2023). Soil microbiomes and one health. Nat. Rev. Microbiol. 21, 6–20. doi: 10.1038/s41579-022-00779-w 35999468

[B3] Bao S. D. (2000). “ Soil and agricultural chemistry analysis (third version),” in China Agriculture Press Beijing. Beijing, China: China Agriculture Press.

[B4] Berendsen R. L. Pieterse C. M. J. Bakker P. A. H. M. (2012). The rhizosphere microbiome and plant health. Trends Plant Sci. 17, 478–486. doi: 10.1016/j.tplants.2012.04.001 22564542

[B5] Brooker R. W. Bennett A. E. Cong W. F. Daniell T. J. George T. S. Hallett P. D. . (2015). Improving intercropping: a synthesis of research in agronomy, plant physiology and ecology. New Phytol. 206, 107–117. doi: 10.1111/nph.13132 25866856

[B6] Cappelli S. L. Domeignoz-Horta L. A. Loaiza V. Laine A.-L. (2022). Plant biodiversity promotes sustainable agriculture directly and via belowground effects. Trends Plant Sci. 27, 674–687. doi: 10.1016/j.tplants.2022.02.003 35279365

[B7] Chamkhi I. Cheto S. Geistlinger J. Zeroual Y. Kouisni L. Bargaz A. . (2022). Legume-based intercropping systems promote beneficial rhizobacterial community and crop yield under stressing conditions. Ind. Crops Prod. 183, 114958. doi: 10.1016/j.indcrop.2022.11495842574925

[B8] Chen W. Modi D. Picot A. (2023). Soil and phytomicrobiome for plant disease suppression and management under climate change: A review. Plants 12, 2736. doi: 10.3390/plants12142736 37514350 PMC10384710

[B9] Chen Y. Du J. Li Y. Tang H. Yin Z. Yang L. . (2022). Evolutions and managements of soil microbial community structure drove by continuous cropping. Front. Microbiol. 13, 839494. doi: 10.3389/fmicb.2022.839494 35295291 PMC8920486

[B10] Chen Z. Wang W. Chen L. Zhang P. Liu Z. Yang X. . (2024). Effects of pepper–maize intercropping on the physicochemical properties, microbial communities, and metabolites of rhizosphere and bulk soils. Environ. Microbiome 19, 108. doi: 10.1186/s40793-024-00653-7 39696399 PMC11657000

[B11] Cuartero J. Pascual J. A. Vivo J.-M. Özbolat O. Sánchez-Navarro V. Egea-Cortines M. . (2022). A first-year melon/cowpea intercropping system improves soil nutrients and changes the soil microbial community. Agric. Ecosyst. Environ. 328, 107856. doi: 10.1016/j.agee.2022.10785642574925

[B12] Curtright A. J. Tiemann L. K. (2021). Intercropping increases soil extracellular enzyme activity: A meta-analysis. Agric. Ecosyst. Environ. 319, 107489. doi: 10.1016/j.agee.2021.10748942574925

[B13] Dang K. Gong X. Zhao G. Wang H. Ivanistau A. Feng B. (2020). Intercropping alters the soil microbial diversity and community to facilitate nitrogen assimilation: A potential mechanism for increasing proso millet grain yield. Front. Microbiol. 11, 601054. doi: 10.3389/fmicb.2020.601054 33324383 PMC7721675

[B14] Gao H. Tian G. Khashi u Rahman M. Wu F. (2022). Cover crop species composition alters the soil bacterial community in a continuous pepper cropping system. Front. Microbiol. 12, 789034. doi: 10.3389/fmicb.2021.789034 35046916 PMC8762165

[B15] Gao J. Zhang F. (2023). Influence of companion planting on microbial compositions and their symbiotic network in pepper continuous cropping soil. J. Microbiol. Biotechnol. 33, 760–770. doi: 10.4014/jmb.2211.11032 37072683 PMC10331949

[B16] Gong X. Wang X. Dang K. Zhang Y. Ji X. Long A. . (2024). Nitrogen availability of mung bean in plant-soil system and soil microbial community structure affected by intercropping and nitrogen fertilizer. Appl. Soil Ecol. 203, 105692. doi: 10.1016/j.apsoil.2024.10569242574925

[B17] Havre G. N. (1961). The flame photometric determination of sodium, potassium and calcium in plant extracts with special reference to interference effects. Anal. Chim. Acta 25, 557–566. doi: 10.1016/S0003-2670(01)81614-7

[B18] Hernández-Pérez T. Gómez-García M. D. R. Valverde M. E. Paredes-López O. (2020). Capsicum annuum (hot pepper): An ancient Latin-American crop with outstanding bioactive compounds and nutraceutical potential. A review. Compr. Rev. Food Sci. Food Saf. 19, 2972–2993. doi: 10.1111/1541-4337.12634 33337034

[B19] Johnson J. L. Temple K. L. (1964). Some variables affecting the measurement of “Catalase activity” in soil. Soil Sci. Soc Am. J. 28, 207–209. doi: 10.2136/sssaj1964.03615995002800020024x

[B20] Kamali-Sarvestani S. Mostowfizadeh-Ghalamfarsa R. Salmaninezhad F. Cacciola S. O. (2022). Fusarium and neocosmospora species associated with rot of cactaceae and other succulent plants. J. Fungi 8, 364. doi: 10.3390/jof8040364 35448595 PMC9024871

[B21] Lai H. Gao F. Su H. Zheng P. Li Y. Yao H. (2022). Nitrogen distribution and soil microbial community characteristics in a legume–cereal intercropping system: A review. Agronomy 12, 1900. doi: 10.3390/agronomy1208190030654563

[B22] Liu C. Wang X. Li X. Yang Z. Dang K. Gong X. . (2024). Effects of intercropping on rhizosphere microbial community structure and nutrient limitation in proso millet/mung bean intercropping system. Eur. J. Soil Biol. 122, 103646. doi: 10.1016/j.ejsobi.2024.10364642574925

[B23] Małachowska-Jutsz A. Matyja K. (2019). Discussion on methods of soil dehydrogenase determination. Int. J. Environ. Sci. Technol. 16, 7777–7790. doi: 10.1007/s13762-019-02375-730311153

[B24] Mao T. Jiang X. (2021). Changes in microbial community and enzyme activity in soil under continuous pepper cropping in response to Trichoderma hamatum MHT1134 application. Sci. Rep. 11, 21585. doi: 10.1038/s41598-021-00951-x 34732764 PMC8566488

[B25] Murphy J. Riley J. P. (1962). A modified single solution method for the determination of phosphate in natural waters. Anal. Chim. Acta 27, 31–36. doi: 10.1016/S0003-2670(00)88444-5

[B26] Nelson D. W. Sommers L. E. (1980). Total nitrogen analysis of soil and plant tissues. J. Assoc. Off. Analytical Chem. 63, 770–778. doi: 10.1093/jaoac/63.4.770

[B27] Olsen S. R. Sommers L. E. (1983). “ Phosphorus,” in Methods of Soil Analysis. (Beijing, China: China Agriculture Press), 403–430.

[B28] Peng Z. Liang C. Gao M. Qiu Y. Pan Y. Gao H. . (2022). The neglected role of micronutrients in predicting soil microbial structure. NPJ Biofilms Microbiomes 8, 103. doi: 10.1038/s41522-022-00363-3 36575178 PMC9794713

[B29] Sagova-Mareckova M. Omelka M. Kopecky J. (2023). The golden goal of soil management: disease-suppressive soils. Phytopathology 113, 741–752. doi: 10.1094/phyto-09-22-0324-kd 36510361

[B30] Spooren J. van Bentum S. Thomashow L. S. Pieterse C. M. J. Weller D. M. Berendsen R. L. (2024). Plant-driven assembly of disease-suppressive soil microbiomes. Annu. Rev. Phytopathol. 62, 1–30. doi: 10.1146/annurev-phyto-021622-100127 38857541

[B31] Tabatabai M. A. Bremner J. M. (1972). Assay of urease activity in soils. Soil Biol. Biochem. 4, 479–487. doi: 10.1016/0038-0717(72)90064-8

[B32] Tan G. Liu Y. Peng S. Yin H. Meng D. Tao J. . (2021). Soil potentials to resist continuous cropping obstacle: Three field cases. Environ. Res. 200, 111319. doi: 10.1016/j.envres.2021.111319 34052246

[B33] Thomas R. L. Sheard R. W. Moyer J. R. (1967). Comparison of conventional and automated procedures for nitrogen, phosphorus, and potassium analysis of plant material using a single digestion. Agron. J. 59, 240–243. doi: 10.2134/agronj1967.00021962005900030010x

[B34] Varley J. A. (1966). Automatic methods for the determination of nitrogen, phosphorus and potassium in plant material. Analyst 91, 119–126. doi: 10.1039/an9669100119

[B36] Wang M. M. Crous P. W. Sandoval-Denis M. Han S. L. Liu F. Liang J. M. . (2022). Fusarium and allied genera from China: species diversity and distribution. Persoonia Mol. Phylogeny Evol. Fungi 48, 1–53. doi: 10.3767/persoonia.2022.48.01 PMC10792286 38234691

[B35] Wang K. Lu Q. Dou Z. Chi Z. Cui D. Ma J. . (2024). A review of research progress on continuous cropping obstacles. Front. Agric. Sci. Eng. 11. doi: 10.15302/J-FASE-2024543

[B37] Wu X. Rensing C. Han D. Xiao K.-Q. Dai Y. Tang Z. . (2022). Genome-resolved metagenomics reveals distinct phosphorus acquisition strategies between soil microbiomes. mSystems 7, e01107-21. doi: 10.1128/msystems.01107-21 35014868 PMC8751388

[B38] Yang J. Duan Y. Liu X. Sun M. Wang Y. Liu M. . (2022). Reduction of banana fusarium wilt associated with soil microbiome reconstruction through green manure intercropping. Agric. Ecosyst. Environ. 337, 108065. doi: 10.1016/j.agee.2022.10806542574925

[B39] Zhu J. Yang Z. Qin F. Guo J. Zhang T. Miao P. (2023). Soil quality assessment of several kinds of typical artificial forestlands in the inner mongolia basin of the yellow river. Land 12, 1024. doi: 10.3390/land1205102430654563

