# Supplementary material for: Effects of pepper–mung bean intercropping on pepper productivity, nutrient accumulation, and rhizosphere microbial communities under continuous cropping
Source: Front Plant Sci. 2026 Oct 30;17:1987577. doi: 10.3389/fpls.2026.1987577 (PMC13759735; doi:10.3389/fpls.2026.1987577)
Supplement: Supplementary file 1 [file DataSheet1.zip › Supplementerary materials.docx]

Supplementary materials

**Table S1** Effects of pepper–mung bean intercropping on growth characteristics of pepper plants at three developmental stages

| Growth stage | Teatment | Plant height（cm） | Stem thick（mm） |
| --- | --- | --- | --- |
| Seedling stage | MP | 27.13±1.51a | 6.21±0.56a |
|  | IP | 27.78±2.29a | 6.85±0.95a |
| Peak flowering Stage | MP | 44.52±4.15a | 11.69±1.78a |
|  | IP | 45.18±3.01a | 12.55±0.96a |
| Peak fruiting stage | MP | 61.50±3.43a | 16.25±1.50a |
|  | IP | 62.51±3.93a | 16.01±1.41a |

Plant height and stem diameter were measured at seedling, peak flowering, and peak fruiting stages. MP, pepper monocropping; IP, pepper in pepper–mung bean intercropping system. Data represent mean ± SD (n = 5). Different lowercase letters indicate significant differences among treatments (*P* < 0.05).

**Figure S1.** Effects of pepper–mung bean intercropping on pepper growth.

**Figure S2.** The composition of rhizosphere fungal communities varies. The fungal community composition at the genus level.

**Figure S3.** The composition of rhizosphere bacterial communities varies. The bacterial community composition at the genus level.
